# Acute Free-Iron Exposure Does Not Explain the Impaired Haemorheology Associated with Haemochromatosis

**DOI:** 10.1371/journal.pone.0146448

**Published:** 2016-01-07

**Authors:** Antony P. McNamee, Surendran Sabapathy, Indu Singh, Jarod Horobin, Janelle Guerrero, Michael J. Simmonds

**Affiliations:** 1 School of Allied Health Sciences, Griffith University, Queensland, Australia; 2 Menzies Health Institute Queensland, Griffith University, Queensland, Australia; 3 School of Medical Sciences, Griffith University, Queensland, Australia; Université Claude Bernard Lyon 1, FRANCE

## Abstract

**Introduction:**

Given the severity of the current imbalance between blood donor supply and recipient demand, discarded blood drawn from the routine venesections of haemochromatosis (HFE-HH) patients may serve as a valuable alternative source for blood banks and transfusion. We investigated whether functional or biochemical differences existed between HFE-HH and control blood samples, with particular focus upon the haemorheological properties, to investigate the viability of venesected blood being subsequently harvested for blood products.

**Methods:**

Blood samples were collected from HFE-HH patients undergoing venesection treatment (n = 19) and healthy volunteers (n = 8). Moreover, a second experiment investigated the effects of a dose-response of iron (0, 40, 80, 320 mM FeCl_3_) on haemorheology in healthy blood samples (n = 7). Dependent variables included basic haematology, iron status, haematocrit, red blood cell (RBC) aggregation (native and standardised haematocrit) and “aggregability” (RBC tendency to aggregate in a standard aggregating medium; 0.4 L/L haematocrit in a Dx70), and RBC deformability.

**Results:**

Indices of RBC deformability were significantly decreased for HFE-HH when compared with healthy controls: RBC deformability was significantly decreased at 1–7 Pa (p < 0.05), and the shear stress required for half maximal deformability was significantly increased (p < 0.05) for HFE-HH. RBC aggregation in plasma was significantly increased (p < 0.001) for HFE-HH, although when RBC were suspended in plasma-free Dx70 no differences were detected. No differences in RBC deformability or RBC aggregation/aggregability were detected when healthy RBC were incubated with varying dose of FeCl_3_.

**Conclusion:**

HFE-HH impairs the haemorheological properties of blood; however, RBC aggregability was similar between HFE-HH and controls when cells were suspended in a plasma-free medium, indicating that plasma factor(s) may explain the altered haemorheology in HFE-HH patients. Acute exposure to elevated iron levels does not appear (in isolation) to account for these differences. Further consideration is required prior to utilising routine venesection blood for harvesting RBC concentrates due to the potential risk of microvascular disorders arising from impaired haemorheology.

## Introduction

High-volume blood transfusion has become standard practice and is essential for the survival of patients with various cancers, severe burns and serious trauma. Blood donor participation and compliance rates, however, indicate an imbalance between supply and demand of blood products, emphasising the necessity for alternate strategies to increase blood stores [1]. One such alternative may include salvaging the blood typically discarded during routine venesection treatment of haemochromatosis patients. Hereditary haemochromatosis (HFE-HH) is a recessive autosomal genetic disorder characterised by excessive accumulation of stored iron (i.e., “iron overload”) in the body [2]. Iron overload occurs in individuals with HFE-HH due to gene mutations that result in impaired regulation of iron transport across the gastrointestinal tract. The excessive iron uptake is initially buffered by transferrin; however, once transferrin binding capacity is saturated, plasma iron concentration increases [3, 4]. The onset of HFE-HH, is typically a slow process, with no obvious symptoms presenting until the fifth-to-sixth decades of life. Comorbidities associated with increased plasma iron concentration and tissue oxidative damage caused by HFE-HH include arthritis and diabetes mellitus [5].

Effective management of HFE-HH currently involves either regular venesection (blood-letting) or pharmacological interventions, such as chelation therapy, that minimise iron accumulation. The primary limitations of pharmacological therapies include the financial burden to the health system, and the adverse effects associated with iron chelation agents; consequently, venesection is often the preferred primary treatment of HFE-HH [6]. Routine venesection of approximately one unit of blood (~7 ml blood per kg body mass; ~450 mL) is highly effective at decreasing plasma iron concentration and reducing mortality rate of individuals with HFE-HH [7, 8]. While the venesected blood of HFE-HH patients is currently discarded in many territories, it is plausible that this blood represents a valuable source of, and for, blood product development. Given that one unit of packed red blood cells cost approximately US$200, and current Australian donor rates represent only 3.5% of the population, the discarded blood drawn from individuals with HFE-HH could account for approximately 25% of current donations, and may possibly serve as a valuable alternative source to alleviate the imbalance between blood donor supply and recipient transfusion demand [1, 9, 10].

Elevated plasma ferritin concentration and increased oxidative stress within blood (key factors associated with HFE-HH) are thought to reversibly impact the physical properties of RBC. Current findings indicate that increased plasma iron concentration or blood drawn from individuals with HFE-HH have altered RBC morphology [11] in addition to functional differences including elevated platelet aggregation [12]. Several macro- and micro-vascular complications are associated with HFE-HH, suggestive of impaired haemorheology and/or microcirculation, including endothelial dysfunction [13, 14] and an independent risk factor for proliferative diabetic retinopathy [15]. Intriguingly, very little is known regarding the haemorheological properties of HFE-HH. Therefore, the primary aim of the present study was to determine whether haemorheological differences exist between blood samples obtained from individuals with HFE-HH when compared with healthy controls. To further elucidate whether increased free iron concentration–typical of HFE-HH–influences blood rheology, the haemorheological properties of blood were examined following the incubation of RBC from healthy donors with varied concentrations of iron chloride (FeCl_3_).

## Materials and Methods

### Protocol–Experiment 1

Twenty-seven individuals volunteered to participate in the present study after providing witnessed and written informed consent. Participants were either individuals with HFE-HH (*n* = 19; 60 ± 14 yr; 1 female) recruited via advertisements placed at a local accredited pathology clinic (SNP Pathology, Queensland) or age-matched healthy controls (CON; *n* = 8; 56 ± 18 yr; 0 females). Individuals with HFE-HH were undergoing new/routine venesection treatment and were not currently using medications known to influence haemorheology, and did not report known cardiovascular, metabolic or neurological comorbidities. Blood was collected from the antecubital portion of the cephalic vein for all participants by an experienced phlebotomist into tubes containing 7.2 mg of K2-EDTA and examined within 4 hours of collection for biochemical and rheological properties. The experimental protocols were reviewed and approved by the Griffith University Human Research and Ethics Committee (Protocol: MSC/16/14/HREC). All reagents were sourced from Sigma Aldrich CO LLC (Sydney Australia) unless otherwise noted.

#### Haematology and biochemistry measurements

Haematocrit (confirmed against the micro-haematocrit method; L/L), haemoglobin concentration (g/L), RBC count (x 10^12^/L), mean cell volume (MCV; fL), mean cell haemoglobin (MCH; pg), and mean cell haemoglobin concentration (MCHC; g/L) were obtained using a haematology analyser (Coulter Act5Diff, Beckman Coulter, Brea, CA). Serum ferritin concentration (ng/mL) of HFE-HH samples was determined via a commercial pathology laboratory (Cobas Integra 800, Roche Diagnostics Ltd., Switzerland).

#### RBC deformability measurements

RBC deformability was measured using an ektacytometer (Rheoscan-D200, Sewon Meditech. Inc., Seoul, Korea) operating at 37 ± 1°C. Homologous RBC (6 μL) were suspended in 600 μL of a standardised solution (dextran 70 in phosphate buffered saline–PBS; viscosity = 30 MPa·s at 37°C, pH = 7.4, 290 mOsmol/kg) and stored at 37 ± 1°C until analysis within 20 min. Immediately prior to measurement, the RBC suspension was transferred into a parallel-plate flow channel (with a height of ∼200μm) that was also stored at 37 ± 1°C. The RBC suspension was then subjected to varied shear stresses ranging from 0 Pa to ∼25 Pa. A low-power laser beam projected through the channel generated a diffraction pattern that was circular for cells at rest and became progressively ellipsoidal as RBC deformed. Diffraction patterns were captured by an integrated digital camera and analysed by fitting an ellipse to the image. An elongation index (EI) of the diffraction pattern was calculated using the following equation: EI = (A–B)/(A + B), where A is the length of the major axis and B is the length of the minor axis of the ellipse. The raw EI-shear stress data provided by the ektacytometer were transferred into a commercial analytical software program (Prism 6, Graphpad Software Inc, La Jolla, CA), where a non-linear curve was fit to the raw EI data within the range of 1.0–25.0 Pa. The subsequent curve fit was used to derive discrete EI across a discrete range of shear stress (0.5, 1.0, 1.5, 3.0, 5.0, 10.0, 15.0 and 20.0 Pa). Moreover, the non-linear curve-fitting algorithm enabled the calculation of the following indices: i. the shear stress required for half of maximal EI (SS_1/2_), and; ii. the maximum EI at infinite shear stress (EI_max_) [16]. Decreased RBC deformability is associated with an increased SS_1/2_ and decreased EI_max_, and the ratio between these two indices (i.e., SS_1/2_:EI_max_) increases with decreased RBC deformability.

#### RBC aggregation measurements

RBC aggregation was determined using a cone-plate shearing system (Myrenne RBC Aggregometer, Myrenne GmbH, Roetgen, Germany). Red blood cell aggregation was measured for two conditions: i. blood samples at native haematocrit; ii. blood samples adjusted to 0.4 L/L haematocrit in autologous plasma. Aggregability of RBC was also determined for samples washed twice in PBS, washed once with a standard aggregating solution (“DX70”; 3% dextran 70 in phosphate buffered saline–PBS–pH = 7.4, 290 mOsmol/kg) and resuspended in DX70 at 0.4 L/L haematocrit.

Blood suspensions (50 μL) were loaded between the cone and the plate shearing system and light transmission was measured. Two values that increase with enhanced RBC aggregation were determined: “M_0_”, the degree of RBC aggregation at stasis within 10 seconds following a cessation of an applied high shear (600 s^-1^); and “M_1_”, the degree of RBC aggregation at a low shear (3 s^-1^) within 10-s following the applied high shear (600 s^-1^). M_0_ and M1 were measured in duplicate and the average value was reported.

### Experiment 2 Protocol–iron incubated independent variable

Whole blood samples from seven healthy donors were centrifuged at 1400 *g* for 10 min before the plasma and the buffy white coat were separated from RBC, and plasma was stored for subsequent use. Packed cells were then washed twice with PBS, and then divided into four aliquots for subsequent incubations with solutions of varied concentration of FeCl_3_. Stock solution of FeCl_3_ (Iron (III) chloride) was serially diluted in PBS to produce 40, 80, and 320 μM solutions. RBC were then suspended in the discrete FeCl_3_ solutions at 0.1 L/L haematocrit, and also in PBS (0.1 L/L haematocrit, 0μM) as a control. Aliquots were incubated at 37°C for 60 min before being washed twice in PBS. Washed RBC were then resuspended in autologous plasma at 0.4 L/L haematocrit, and measurements of RBC aggregation and RBC deformability were performed using the methodology described for Experiment 1. All measurements were performed in duplicate and the average used for statistical analyses.

#### Statistical analysis

Normality of the data was examined using the Shapiro-Wilk test with visual inspection for kurtosis and skew of the data. Greenhouse-Geisser corrections were applied, when necessary, where an inequality of variance was detected. Differences in the mean values for each group were compared using independent–samples *t*–tests. Data from each FeCl_3_ solution in Experiment 2 were compared using a one-way ANOVA with repeated measures, to determine whether significant differences in the means existed (Prism 6, Graphpad Software Inc., La Jolla, CA). Data are reported as mean ± standard error unless otherwise stated.

## Results

### Experiment 1 –HFE-HH vs Control

#### Haematology

Clinical haematology indices and plasma ferritin concentration for HFE-HH and healthy controls are presented in Table 1. Haematocrit was not significantly different between HFE-HH and control. Further analyses demonstrated that regular venesection was effective at decreasing haematocrit, given that when HFE-HH were clustered into “new cases” (no prior venesection treatment within 12-mo; n = 5) or those receiving routine venesection (n = 14), new patients presented with significantly elevated haematocrit (0.45 ± 0.03) when compared with regular venesection HFE-HH patients (0.39 ± 0.02; p < 0.001). The MCH was increased for HFE-HH (p = 0.02) when compared with controls, although both groups were within normal ranges.

**Table 1:** Venous blood characteristics of HFE-HH and control samples.

	CON (*n* = 8)	HFE-HH (*n* = 17)	Normal Range
Haematocrit (L/L)	0.41 ± 0.04	0.41 ± 0.05	0.40–0.54
RBC count (x 10^12^/L)	4.88 ± 0.47	4.49 ± 0.59*	4.5–6.5
Haemoglobin (g/L)	137 ± 16	137 ± 17	115–180
MCV (fL)	86 ± 3	90 ± 4	80–100
MCH (pg)	27.5 ± 2.5	30.7 ± 1.7*	27–32
MCHC (g/L)	331 ± 8	341 ± 6	300–350
Ferritin (ng/mL)	Within normal range	328 ± 274	20–200

Data are mean ± standard deviation. CON: healthy controls. HFE-HH: patients with haemochromatosis. RBC: red blood cell. MCV: mean corpuscular volume. MCH: mean corpuscular haemoglobin. MCHC: mean corpuscular haemoglobin concentration.

*, HFE-HH significantly different compared with CON (p < 0.05).

#### RBC deformability

**Fig 1:**
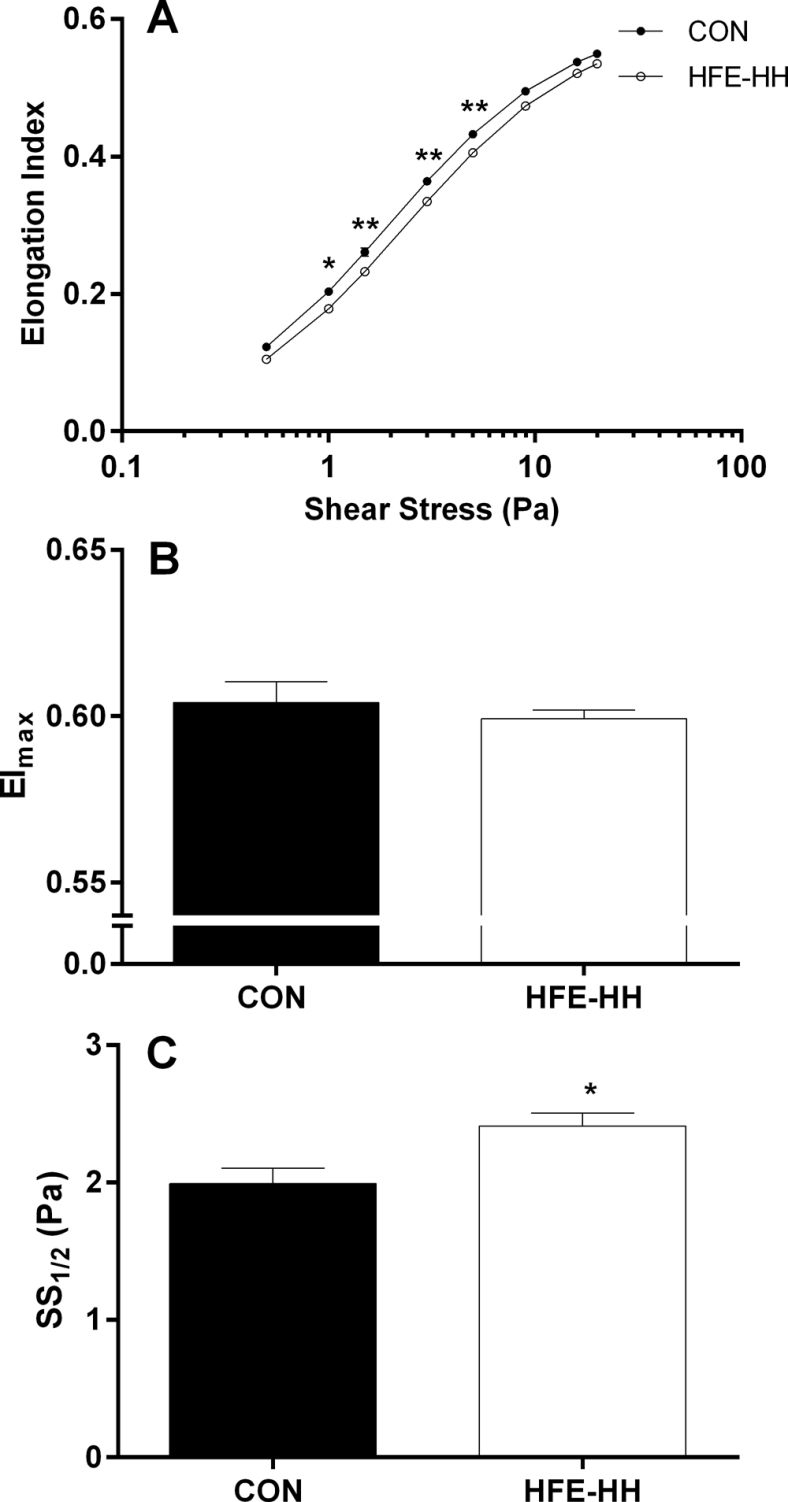
Red cell deformability (Elongation Index; Panel A) was decreased at discrete shear stresses for haemochromatosis (HFE-HH) when compared with healthy controls (CON). Maximal Elongation Index (EI_max_) was not different between groups (Panel B); however, the shear stress required for half-maximal elongation index (SS_1/2_) was significantly increased for HFE-HH (Panel C). *, p < 0.05. **, p < 0.01.

Indices of RBC deformability for HFE-HH and Controls are illustrated in Fig 1. The EI (i.e., cell deformability) response to shear stress (Fig 1A) was typically sigmoidal for both groups; however, a right-shift in this relation was observed for HFE-HH. Indeed, EI was significantly decreased at all shears within the range of 1.0–5.0 Pa (p < 0.05) for HFE-HH, although there were no significant differences between groups at higher shear stress. While the maximal theoretical RBC deformability (i.e., EI_max_; Fig 1B) was not different between groups, SS_1/2_ was increased by 20% (Fig 1C) for HFE-HH when compared with control (p < 0.05).

#### RBC aggregation

**Fig 2:**
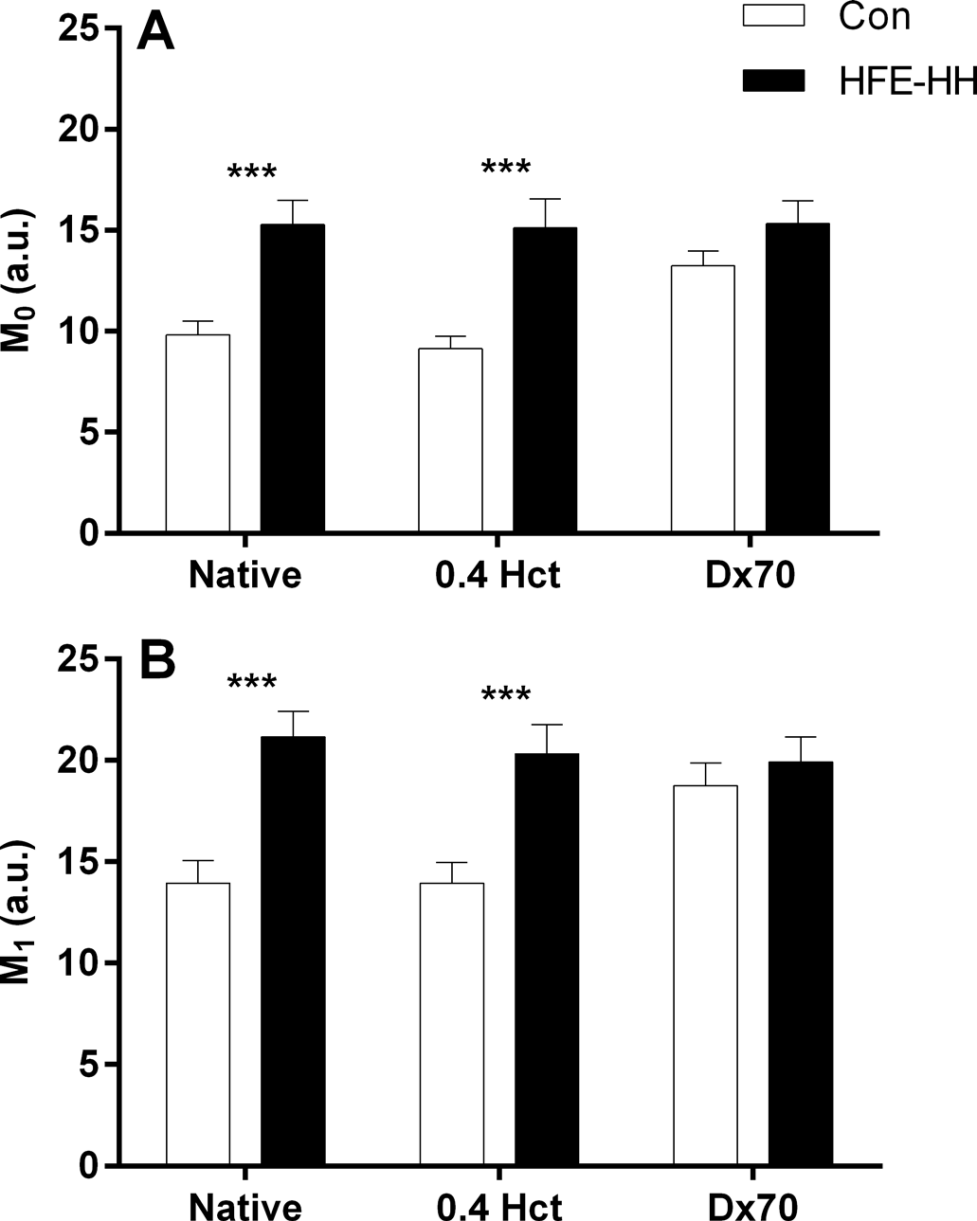
Red cell aggregation for haemochromatosis (HFE-HH) patients and healthy controls (Con) measured in plasma suspensions at native and a standardised (0.4 L/L) haematocrit, and red cell aggregability in a standard aggregating medium (Dx70). Red cell aggregation was increased for HFE-HH when compared with Con while at stasis (“M_0_”; Panel A) at native and standardised haematocrit. The same observation was made for aggregation measures at low-shear (“M_1_”; Panel B). Red cell aggregability was not different between groups at stasis or low-shear. ***, HFE-HH significantly increased compared with Con, p < 0.001.

Indices that characterise aggregation of RBC in plasma at either native or standardised haematocrit, and in a plasma-free aggregating solution (i.e., Dx70), are illustrated in Fig 2. When RBC were suspended in autologous plasma at native haematocrit, RBC aggregation at stasis (M_0_; Fig 2A) and at low shear (M_1_; Fig 2B) was significantly increased in HFE-HH when compared with control (p < 0.001). To standardise for the pro-aggregating effects of elevated haematocrit, samples were adjusted to 0.4 L/L haematocrit, where it was also found that RBC aggregation at stasis (M_0_) and at low shear (M_1_) remain elevated in HFE-HH compared with control (p < 0.001). No differences were observed for RBC aggregability when RBC were suspended in plasma-free dextran 70 (i.e., Dx70).

### Experiment 2 –Iron incubation

#### RBC Deformability

**Fig 3:**
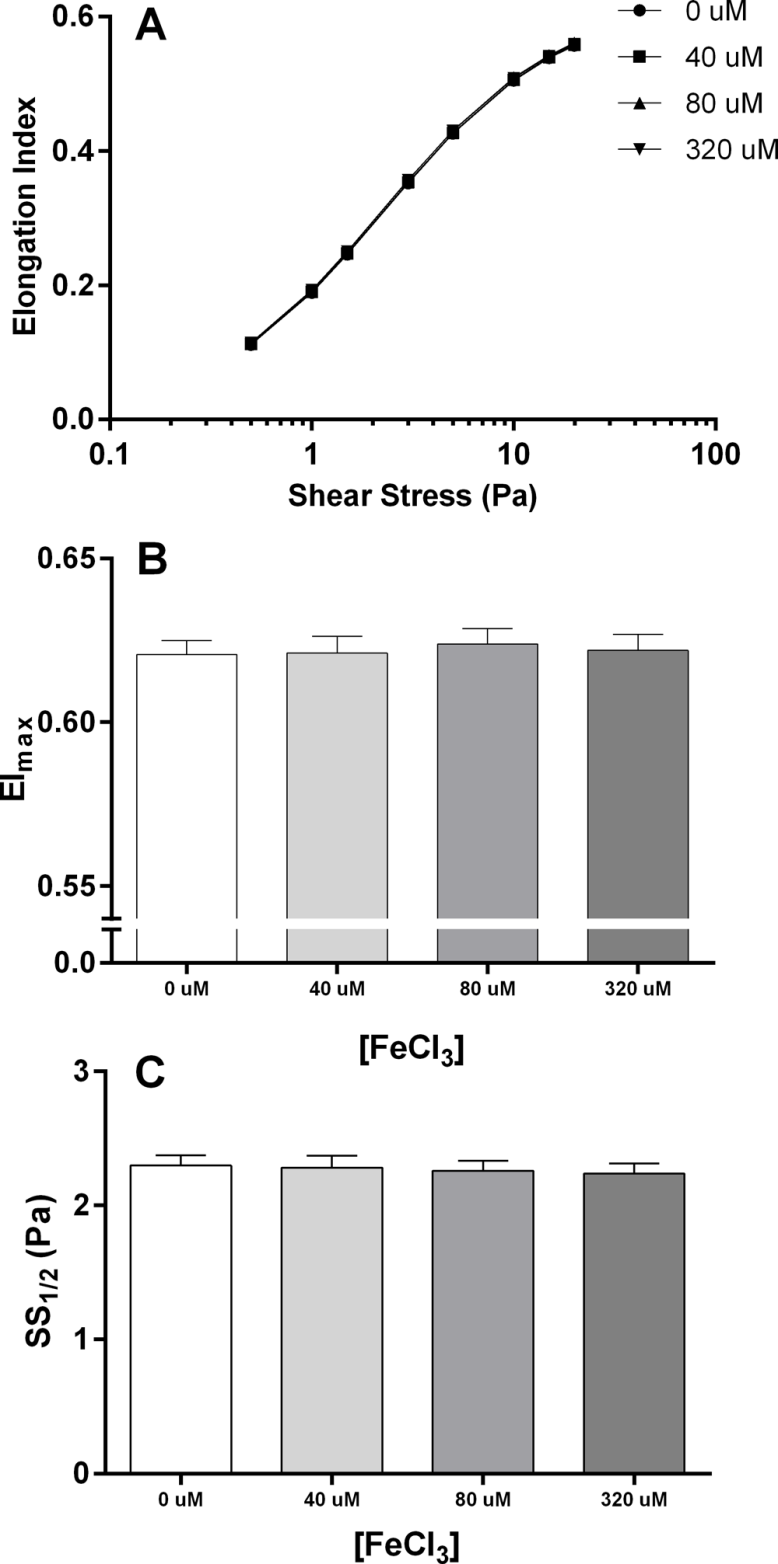
The Elongation Index (EI) of RBC in autologous plasma at varying shear stresses (Panel A) following an incubation with varying concentration of FeCl_3_ (0, 40, 80, 320 uM). The varied iron incubated conditions did not influence the maximal EI (EI_max_; Panel B) or the amount of shear stress required for half maximal EI (SS_half_; Panel C).

The deformability (EI) of RBC exposed to varying shear stresses following incubation with varying concentrations of FeCl_3_ (0, 40, 80, 320 μM) is illustrated in Fig 3A. One hour of incubation with FeCl_3_, irrespective of dose, did not influence EI at any shear stress (F = 2.01, p = 0.15). Consequently, the dose of FeCl_3_ incubation did not significantly affect EI_max_ (F = 0.54, p = 0.53; Fig 3B) or SS_1/2_ (F = 0.50, p = 0.69; Fig 3C).

#### RBC aggregation

**Fig 4:**
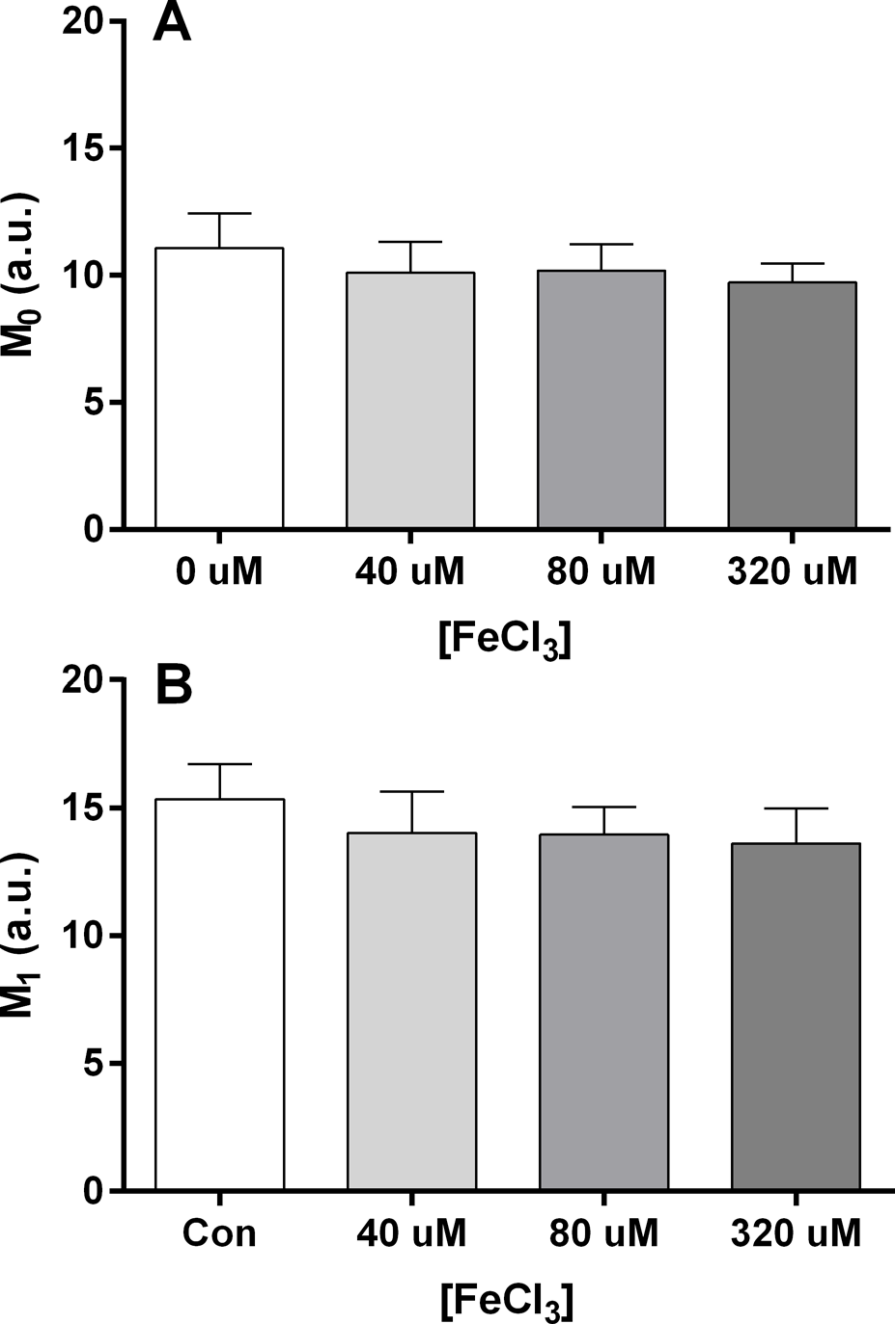
Aggregation of RBC suspensions in autologous plasma following incubation with various concentration of FeCl_3_. Incubation with FeCl_3_ did not influence the RBC aggregation at stasis (M_0_; Panel A) or at low-shear (M_1_; Panel B).

Indices that describe the aggregation of RBC suspended in autologous plasma following incubation with varying concentrations of FeCl_3_ (0, 40, 80, 320 uM) are shown in Fig 4. Varying FeCl_3_ concentration had no significant influence on RBC aggregation at stasis (M_0_: F = 0.27, p = 0.85; Fig 4A) or at low-shear (M_1_: F = 0.33, p = 0.81; Fig 4B).

## Discussion

The salient findings of the present study are: i. RBC deformability was significantly decreased in HFE-HH when compared with control; ii. RBC aggregation was significantly increased at stasis and under low shear for HFE-HH when compared with control, independent of Hct; iii. RBC aggregability in a standardised plasma-free suspending medium was not different between groups and; iv. Incubation of RBC with iron did not significantly influence RBC deformability or RBC aggregation. The collective findings of the present study indicate that HFE-HH induces haemorheological impairments that are independent of haematocrit, and are emphasised in autologous plasma. When RBC from HFE-HH were suspended in a plasma-free medium, RBC aggregability was not different to control, suggesting that plasma factor(s) explain the haemorheological derangement in HFE-HH. The experimental findings also suggest that acute elevations of free iron do not, in isolation, explain the impaired haemorheology that characterises HFE-HH patients. Further consideration is required prior to using RBC concentrate, drawn from routine venesection treatment of HFE-HH, as a donor source for transfusion patients.

To our knowledge, the present is the first report of impaired RBC deformability in HFE-HH, although RBC morphological impairments have been noted due to high iron concentration [17]. Iron has a dose-dependent role in haemorheological health: while conditions classically associated with iron-overload are associated with impaired haemorheology, iron supplementation may correct haemorheological parameters in iron-deficient anaemia [18, 19]. The lipid bilayer of RBC membranes is susceptible to oxidative damage from free radicals, and the Na,K ATPase of RBC are modulated by FeCl_3_ [20], thus elevated free iron may explain altered haemorheology through promotion of reactive species generation [21, 22] as well as altered cell volume modulated via ion channels [20]. In the present study, HFE-HH was associated with a decreased elongation index (i.e., RBC deformability) at low-to-moderate shear stress and an increased (i.e., impaired) SS_1/2_: these findings collectively indicate that RBC from HFE-HH patients were less deformable/more rigid than controls. The functional consequences of such a decrease in RBC deformability include heightened risk for microvascular disease and organ damage, given that nutrient delivery to distal tissues may be impaired [23, 24]. Previous studies have demonstrated that HFE-HH is closely associated with venous disease [25], and iron deposition in various tissues is closely associated with organ damage and failure [26]. Thus, altered RBC deformability may be implicit in some of the co-morbidities that accompany HFE-HH.

It was also observed that aggregation of RBC suspended in plasma was elevated in patients with HFE-HH. A primary factor that promotes RBC aggregation is the number of RBC in solution (e.g., RBC count; haematocrit) [24] which increases the probability of cell-cell contact, and it was found that RBC count of HFE-HH was significantly decreased (albeit at the lower limit of “normal ranges”) when compared with CON. This decrease in RBC count may be explained due to the frequent and routine venesections of the HFE-HH patients. Differences in haematocrit does not explain the increased RBC aggregation, given that when suspensions were adjusted to a standardised haematocrit (0.4 L/L), RBC aggregation of HFE-HH remained significantly increased when compared with CON. The magnitude of RBC aggregation is far from a simple outcome, however, and is determined by factors intrinsic (e.g., cell membrane charge) and extrinsic (e.g., plasma fibrinogen concentration) to the cell [24]. Previous investigations have demonstrated that structural changes in the RBC membrane may be observed in HFE-HH, including an iron-dependent elongation in cell morphology that is reversible with iron-chelation [27]. Thus, it would be attractive to speculate that such changes to RBC morphology might explain the hyperaggregability of HFE-HH in the present study. Data obtained in the present study may exclude intrinsic causes of increased RBC aggregation in HFE-HH, given that when RBC were suspended in a plasma-free medium (i.e., Dx70), RBC aggregability was not significantly different to control. This finding suggests that plasma factors must explain the increased RBC aggregation in HFE-HH.

The plasma factors that may serve as obvious candidates responsible for elevated RBC aggregation in HFE-HH include fibrinogen, and potentially, iron. While we did not measure plasma fibrinogen, previous large cohort (16,000 to 25,000 patients) studies have failed to demonstrate an association between serum ferritin concentration, or the HFE gene, and increased plasma fibrinogen concentration [28, 29]. In contrast, there is a relative abundance of studies demonstrating associations between chronic inflammation, hypercoagulation (including fibrinogen), and excess iron [30, 31]. Indeed, a common comorbidity of HFE-HH is type 2 diabetes, which is closely associated with impaired haemorheology [32, 33], due in part, to increased fibrinogen and alpha 2-macroglobulin [34]; our patients were reported free of diabetes. Moreover, our experimental data (i.e., Experiment 2) appears to rule out free iron as a candidate, given that when RBC from healthy donors were incubated in both physiological and supra-physiological FeCl_3_ concentrations, even a mild albeit non-significant decrease of RBC aggregation was observed. In light of these findings, it is plausible that while HFE/serum iron may not directly induce increased fibrinogen concentration, the associated low-level inflammation and oxidative stress that is commonly observed in iron-overload conditions may explain, in part, the altered haemorheology.

Incubation of RBC from healthy donors with varying concentrations of iron did not affect RBC deformability. Experiment 2 was implemented to determine whether free iron, in isolation, might explain some of the decreased RBC deformability (and increased RBC aggregation) observed for HFE-HH in Experiment 1. The results of Experiment 2 indicate that free iron, in the absence of other blood constituents (e.g., cells, plasma proteins, etc.) does not influence RBC deformability; to our knowledge, this finding has not previously been reported. Our direct measurement of RBC deformability following incubation with FeCl_3_ extends the recent finding that FeCl_3_ modulated Na,K ATPase of RBC, which presumably would influence cell volume, ion content, and thus would be predicted to determine RBC deformability [20]. It is possible that increased Na,K ATPase activity in response to FeCl_3_ may be offset by opposing factors during incubation studies (e.g., cell ageing; temperature effects etc) and thus result in no net-functional change to RBC deformability; however, it is also likely that differences in incubation period of the present study (60 min) and that of Sousa et al., [20] may explain, in part, these discordant findings. The fact that we did not observe impaired haemorheology with acute iron incubation *in vitro* does not discount the possibility that chronic exposure to iron, or its interaction with other [plasma?] factors promoting oxidative stress [35, 36], *in vivo*, influences RBC aggregation and deformability in HFE-HH.

The clinical context of the present study was based on investigating the potential for discarded HFE-HH venesections to be used as a source for harvesting blood products. As such, venous blood samples were obtained from HFE-HH patients who were undergoing routine venesection treatment. We were able to investigate a small subset of these HFE-HH patients who had not previously undergone routine venesections (n = 5). While it was not a primary aim to investigate differences in haemorheology with respect to duration of treatment, there were data that indicated routine venesection may positively modulate haematological profile (e.g., decreased haematocrit); it is of interest whether haematocrit-sensitive haemorheology (e.g., RBC aggregation) also improves with routine venesection. It is intriguing, therefore, to consider whether other haemorheological factors–e.g., RBC deformability–may also be improved with routine venesection in HFE-HH, although this observation requires further investigation with a larger cohort. With respect to the primary aim, however, the collective findings of Experiment 1 strongly suggest that HFE-HH blood is rheologically different to healthy controls, and further consideration is required prior to utilising routine venesection blood for harvesting RBC concentrate. Given impaired haemorheology is primal to various non-communicable diseases, there is potential for acute microvascular disorders to arise in otherwise healthy individuals that might receive HFE-HH derived RBC concentrate via transfusion.

## References

[1] Masser B, France CR. An evaluation of a donation coping brochure with Australian non-donors. Transfusion and apheresis science: official journal of the World Apheresis Association: official journal of the European Society for Haemapheresis. 2010;43(3):291–7. 10.1016/j.transci.2010.09.017 . 20934386

[2] Pietrangelo A. Hereditary hemochromatosis—a new look at an old disease. The New England journal of medicine. 2004;350(23):2383–97. 10.1056/NEJMra031573 . 15175440

[3] Camaschella C. Understanding iron homeostasis through genetic analysis of hemochromatosis and related disorders. Blood. 2005;106(12):3710–7. 10.1182/blood-2005-05-1857 . 16030190

[4] Montosi G, Donovan A, Totaro A, Garuti C, Pignatti E, Cassanelli S, et al Autosomal-dominant hemochromatosis is associated with a mutation in the ferroportin (SLC11A3) gene. The Journal of clinical investigation. 2001;108(4):619–23. 10.1172/JCI13468 11518736 PMC209405

[5] Swaminathan S, Fonseca VA, Alam MG, Shah SV. The role of iron in diabetes and its complications. Diabetes care. 2007;30(7):1926–33. 10.2337/dc06-2625 . 17429063

[6] Kirking MH. Treatment of chronic iron overload. Clinical pharmacy. 1991;10(10):775–83. Epub 1991/10/01. . 1742962

[7] Adams PC, Barton JC. How I treat hemochromatosis. Blood. 2010;116(3):317–25. 10.1182/blood-2010-01-261875 . 20308595

[8] Niederau C, Fischer R, Sonnenberg A, Stremmel W, Trampisch HJ, Strohmeyer G. Survival and causes of death in cirrhotic and in noncirrhotic patients with primary hemochromatosis. The New England journal of medicine. 1985;313(20):1256–62. 10.1056/NEJM198511143132004 . 4058506

[9] Toner RW, Pizzi L, Leas B, Ballas SK, Quigley A, Goldfarb NI. Costs to hospitals of acquiring and processing blood in the US: a survey of hospital-based blood banks and transfusion services. Applied health economics and health policy. 2011;9(1):29–37. 10.2165/11530740-000000000-00000 . 21174480

[10] Waters JR, Meier HH, Waters JH. An economic analysis of costs associated with development of a cell salvage program. Anesthesia and analgesia. 2007;104(4):869–75. 10.1213/01.ane.0000258039.79028.7c . 17377098

[11] Pretorius E, Lipinski B. Iron alters red blood cell morphology. Blood. 2013;121(1):9 23409279 10.1182/blood-2012-09-454793

[12] Lynch S, Soslau G. Iron levels found in hemochromatosis patients inhibit gamma-thrombin-induced platelet aggregation. Platelets. 2012;23(8):611–6. 10.3109/09537104.2011.634933 . 22111666

[13] Gaenzer H, Marschang P, Sturm W, Neumayr G, Vogel W, Patsch J, et al Association between increased iron stores and impaired endothelial function in patients with hereditary hemochromatosis. J Am Coll Cardiol. 2002;40(12):2189–94. . 12505233 10.1016/s0735-1097(02)02611-6

[14] Lekakis J, Papamicheal C, Stamatelopoulos K, Cimponeriu A, Voutsas A, Vemmos K, et al Hemochromatosis associated with endothelial dysfunction: evidence for the role of iron stores in early atherogenesis. Vasc Med. 1999;4(3):147–8. . 10512594 10.1177/1358836X9900400305

[15] Peterlin B, Globocnik Petrovic M, Makuc J, Hawlina M, Petrovic D. A hemochromatosis-causing mutation C282Y is a risk factor for proliferative diabetic retinopathy in Caucasians with type 2 diabetes. J Hum Genet. 2003;48(12):646–9. 10.1007/s10038-003-0094-3 . 14618419

[16] Baskurt OK, Hardeman MR, Uyuklu M, Ulker P, Cengiz M, Nemeth N, et al Parameterization of red blood cell elongation index—shear stress curves obtained by ektacytometry. Scandinavian journal of clinical and laboratory investigation. 2009;69(7):777–88. Epub 2009/11/26. 10.3109/00365510903266069 . 19929721

[17] Pretorius E. The adaptability of red blood cells. Cardiovascular diabetology. 2013;12:63 10.1186/1475-2840-12-63 23578325 PMC3637111

[18] Ciccoli L, Signorini C, Scarano C, Rossi V, Bambagioni S, Ferrali M, et al Iron release in erythrocytes from patients with beta-thalassemia. Free radical research. 1999;30(5):407–13. . 10342333 10.1080/10715769900300441

[19] Halis H, Bor-Kucukatay M, Akin M, Kucukatay V, Bozbay I, Polat A. Hemorheological parameters in children with iron-deficiency anemia and the alterations in these parameters in response to iron replacement. Pediatric hematology and oncology. 2009;26(3):108–18. 10.1080/08880010902754909 . 19382032

[20] Sousa L, Garcia IJ, Costa TG, Silva LN, Reno CO, Oliveira ES, et al Effects of Iron Overload on the Activity of Na,K-ATPase and Lipid Profile of the Human Erythrocyte Membrane. PLoS One. 2015;10(7):e0132852 10.1371/journal.pone.0132852 26197432 PMC4510300

[21] Comporti M, Signorini C, Buonocore G, Ciccoli L. Iron release, oxidative stress and erythrocyte ageing. Free radical biology & medicine. 2002;32(7):568–76. . 11909691 10.1016/s0891-5849(02)00759-1

[22] Simmonds MJ, Meiselman HJ, Marshall-Gradisnik SM, Pyne M, Kakanis M, Keane J, et al Assessment of oxidant susceptibility of red blood cells in various species based on cell deformability. Biorheology. 2011;48(5):293–304. 10.3233/BIR-2012-0599 . 22433570

[23] Baskurt OK, Meiselman HJ. Blood rheology and hemodynamics. Seminars in thrombosis and hemostasis. 2003;29(5):435–50. 10.1055/s-2003-44551 . 14631543

[24] Simmonds MJ, Meiselman HJ, Baskurt OK. Blood rheology and aging. J Geriatr Cardiol. 2013;10(3):291–301. 10.3969/j.issn.1671-5411.2013.03.010 24133519 PMC3796705

[25] Zamboni P, Tognazzo S, Izzo M, Pancaldi F, Scapoli GL, Liboni A, et al Hemochromatosis C282Y gene mutation increases the risk of venous leg ulceration. Journal of vascular surgery. 2005;42(2):309–14. 10.1016/j.jvs.2005.04.003 . 16102632

[26] Emanuele D, Tuason I, Edwards QT. HFE-associated hereditary hemochromatosis: overview of genetics and clinical implications for nurse practitioners in primary care settings. Journal of the American Association of Nurse Practitioners. 2014;26(3):113–22. 10.1002/2327-6924.12106 . 24574363

[27] Pretorius E, Bester J, Vermeulen N, Lipinski B, Gericke GS, Kell DB. Profound morphological changes in the erythrocytes and fibrin networks of patients with hemochromatosis or with hyperferritinemia, and their normalization by iron chelators and other agents. PloS one. 2014;9(1):e85271 10.1371/journal.pone.0085271 24416376 PMC3887013

[28] Forouhi NG, Harding AH, Allison M, Sandhu MS, Welch A, Luben R, et al Elevated serum ferritin levels predict new-onset type 2 diabetes: results from the EPIC-Norfolk prospective study. Diabetologia. 2007;50(5):949–56. 10.1007/s00125-007-0604-5 . 17333112

[29] Pankow JS, Boerwinkle E, Adams PC, Guallar E, Leiendecker-Foster C, Rogowski J, et al HFE C282Y homozygotes have reduced low-density lipoprotein cholesterol: the Atherosclerosis Risk in Communities (ARIC) Study. Translational research: the journal of laboratory and clinical medicine. 2008;152(1):3–10. 10.1016/j.trsl.2008.05.005 18593631 PMC2587433

[30] Pretorius E, Kell DB. Diagnostic morphology: biophysical indicators for iron-driven inflammatory diseases. Integrative biology: quantitative biosciences from nano to macro. 2014;6(5):486–510. 10.1039/c4ib00025k . 24714688

[31] Lipinski B, Pretorius E, Oberholzer HM, van der Spuy WJ. Interaction of fibrin with red blood cells: the role of iron. Ultrastruct Pathol. 2012;36(2):79–84. 10.3109/01913123.2011.627491 . 22471429

[32] Simmonds MJ, Sabapathy S, Gass GC, Marshall-Gradisnik SM, Haseler LJ, Christy RM, et al Heart rate variability is related to impaired haemorheology in older women with type 2 diabetes. Clin Hemorheol Microcirc. 2010;46(1):57–68. 10.3233/CH-2010-1333 . 20852363

[33] Schmid-Schonbein H, Rieger H, Gallasch G, Schachtner H. Pathological red cell aggregation (clump aggregation). Molecular and electrochemical factors. Bibl Anat. 1977;(16 Pt 2):484–9. . 75007

[34] Zimmermann J, Schramm L, Wanner C, Mulzer E, Henrich HA, Langer R, et al Hemorheology, plasma protein composition and von Willebrand factor in type I diabetic nephropathy. Clin Nephrol. 1996;46(4):230–6. . 8905207

[35] Galaris D, Pantopoulos K. Oxidative stress and iron homeostasis: mechanistic and health aspects. Crit Rev Clin Lab Sci. 2008;45(1):1–23. 10.1080/10408360701713104 . 18293179

[36] Ghoti H, Amer J, Winder A, Rachmilewitz E, Fibach E. Oxidative stress in red blood cells, platelets and polymorphonuclear leukocytes from patients with myelodysplastic syndrome. European journal of haematology. 2007;79(6):463–7. 17976187 10.1111/j.1600-0609.2007.00972.x

